# Effects of nitrogen and precipitation on the life history of spring- and autumn-germinated plants of *Hypecoum erectum* L. (Papaveraceae)

**DOI:** 10.1371/journal.pone.0321259

**Published:** 2025-05-09

**Authors:** Shanlin Yang, Rongrong Cui, Xueying Yang, Kexin Sun, Yuwei Liu, Bing Han, Chunzhi Zhou, Bingyan Liu

**Affiliations:** College of Landscape Architecture, Changchun University, Jilin, China; Tennessee State University, UNITED STATES OF AMERICA

## Abstract

Nitrogen deposition and precipitation are the topics of current global climate change, and also the major environmental factors influencing plant growth. This study utilized the ephemeral plant *H. erectum*, which is distributed in the Gurbantunggut Desert in northwest China, as the experimental material to analyze the influence of nitrogen deposition and water-nitrogen interaction treatment on the phenology, survival rate, plant traits, biomass accumulation, and seed dormancy of spring- and autumn-germinated plants. The research results indicate that increased nitrogen increases the survival rate of *H. erectum* spring- and autumn-germinated plants. There is no significant impact on phenological events. However, plant traits such as leaf number, leaf area, branch number, seed quantity, and biomass accumulation are all reduced. During the growth and development process, more biomass is allocated to reproductive organs, and result in the production of a large number of non-dormant seeds. Therefore, in arid and semi-arid ecosystems, nitrogen deposition plays a crucial role in the survival of plants and the rapid reproduction of offspring. After water-nitrogen interaction treatment, the survival rate of *H. erectum* spring- and autumn-germinated plants significantly increased. The main phenology (leafing date, first flowering date, last flowering date, fruiting date and withering date) were delayed, extending the life cycle of reproductive growth. Biomass accumulation in all organs increased with a same allocation trend, produce a large number of dormant seeds. Therefore, precipitation not only influences the biomass allocation of plants and regulates their nitrogen uptake, changes the growth mechanisms of plants in adverse environments.

## Introduction

Nitrogen is a nutrition for plant growth and one of the most crucial limiting factors as well [1] Increased nitrogen deposition can promote plant growth and increase biomass. However, large amount of nitrogen deposition might reduce the productivity of plants [2,3]. At present, domestic and international research on nitrogen deposition is limited to specific regions or artificially simulated environments. Research on the influence of nitrogen deposition on plants at the population level mainly concentrate on vegetation coverage [4], species diversity, community composition [5], community biomass [6] and the photosynthetic characteristics of plants [7]. Relevant research at the species level merely pertains to soil health, microbial biomass [8,9,10], studies on dry matter nynamics, vegetation structure, and ecological restoration [11,12,13] as well as phenology characteristics [14]. However, a systematic study on the influence of nitrogen deposition on plant life history is lacking, which without a clear elucidation of the specific effects of increased nitrogen deposition on plant growth and its influence on plant resource diversity.

Water is the major limiting factor for plant growth and affecting the transportation of nitrogen in plants [15,16]. Due to climate warming, the precipitation pattern is transforming from regionalization to globalization and simultaneously influencing the absorption and utilization of nitrogen by plants [2]. With an increase in water or nitrogen, plants exhibit synchronous increases in plant traits such as plant height, leaf number, biomass accumulation. Moreover, nitrogen can enhance its promoting effect on plant growth under higher water conditions [17,18,19]. Therefore, the interact between water and nitrogen can influence the growth and development of plants.

In arid and semi-arid areas, water and nitrogen are among the most significant factors limiting plant growth [20]. Over the past 30 years, nitrogen deposition and annual average precipitation in the northwest region of China have increased, and expected that the increase will continued until the end of this century [21,22,23]. Increased nitrogen and precipitation affects the photosynthetic rate of plants and the content of nutrient, thereby change plant growth indicators and species diversity. However, the spring- and autumn-germinated ephemeral plants in the Gurbantunggut Desert in northwest China exhibit rapid growth, high photosynthetic rate, and allocate a large number of biomass to reproductive growth [2]. Therefore, spring- and autumn-germinated ephemeral plants serve as crucial materials for further investigating the response mechanisms of plants to increased nitrogen and precipitation.

Ephemeral plants is a specific plant group in temperate desert. They can rapidly germinate by utilizing the meltwater of winter snow and the precipitation in early spring to complete their life cycle, and large number of ephemeral plants shown two germination season with spring and autumn [24]. Phenology is acknowledged as one of the important traits that contribute significantly to the growth and success of plants [14]. Due to different germination seasons, the life history characteristics of spring- and autumn-germinated plants have significant differences, including life cycle [2], plant size [25], biomass [26], fruit yield [27] and seed characteristics [28]. As climate change and global change intensify gradually, significant changes have occurred in precipitation and nitrogen deposition within temperate desert ecosystems. Therefore, investigating the influences of increased nitrogen and precipitation on the life history of spring- and autumn-germinated plants is of crucial significance for promoting the growth and development of ephemeral plants.

*Hypecoum erectum* L. is an ephemeral plants that grows in autumn- and spring-germinated in the Gurbantunggut Desert, and mainly distributed in Russia, Mongolia and China [29]. In China, it is extensively distributed in the flat sandy areas and fixed sand dunes of the Gurbantunggut Desert [30]. The vegetation coverage in this area reached 18% - 20% in May and it is one of the crucial plants for windbreak and sand fixation in the Gurbantunggut Desert during spring [29]. Research results show that autumn-germinated plants are larger and produce more seeds than spring-germinated plants under natural conditions [16,29]. Based on the difference in biological characteristics of spring- and autumn- germinated plants, we propose the hypothesis that there are differences in the life history of spring- and autumn- germinated plants in response to increased nitrogen and precipitation treatments. To testing this hypothesis, we compared the phenology, survival rate, plant characteristics, biomass accumulation and allocation, and seed characteristics of spring- and autumn- germinated plants with increased nitrogen and precipitation.

## Materials and methods

### Study site

Gurbantunggut Desert is the second largest desert in China. The study area is situated on the southern margin of the Gurbantunggut Desert (84°50′ - 91°20′ E, 49°15′ - 46°50′ N). This region features a typical temperate continental climate, with an annual average temperature of 6.6 °C, an annual average precipitation from 70 to 150 mm, which is mainly concentrated in spring, and an annual evaporation amounting to 3000–3500 mm. In winter, the region is covered with snow, with a stable snow cover time of 95–110 d. Winter precipitation accounts for approximately 30% of the annual [31]. Melting snow in early spring provide favorable water conditions for the growth and development of ephemeral plants in spring [32].

### Experimental material

**Fig 1:**
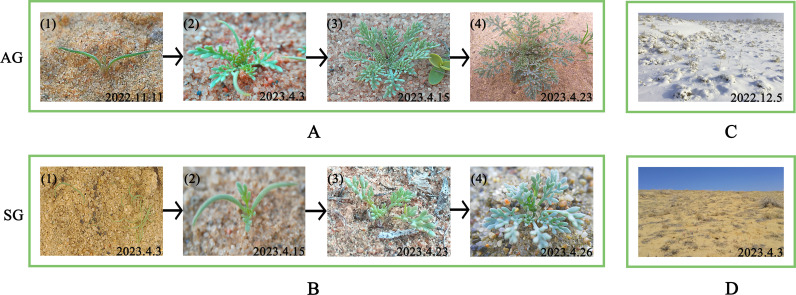
Growth process of spring- and autumn-germinated plants of *Hypecoum erectum.* (A. AG, autumn-germinated plants; B. Autumn-germinated study site; C. SG, spring-germinated plants; D. Spring-germinated study site).

On October 29th, 2022, within the 72 quadrats of the 6 treatments in the autumn-germinated plants field, 15 autumn-germinated plants were randomly marked with copper wires, respectively. On March 30th, 2023, within the 72 quadrats of the 6 treatments in the field of spring-germinated plants, 15 spring-germinated plants were randomly marked with copper wires, respectively. The autumn-germinated plants of *H. erectum* can overwinter under the snow and grow concurrently with the spring-germinated plants in the second spring (Fig 1).

## Experimental design

### Nitrogen increase experiment

**Fig 2:**
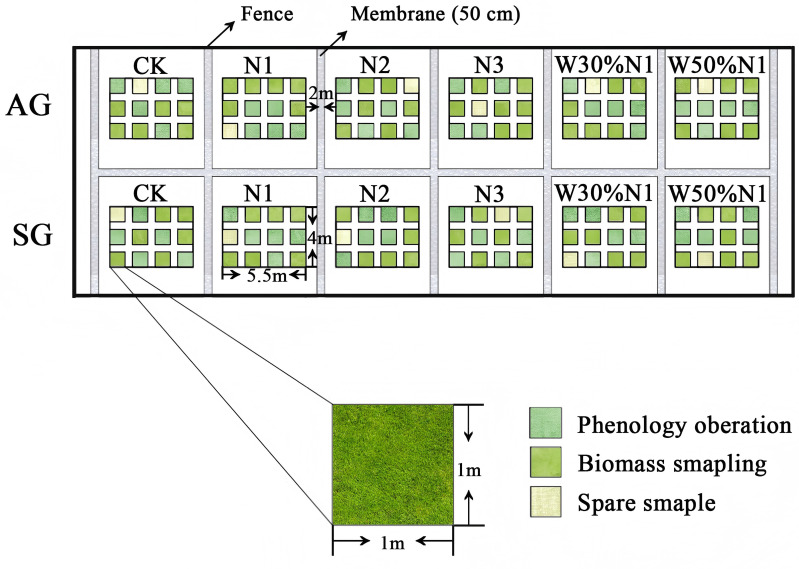
Experimental field for spring- and autumn-germinated plants of *Hypecoum erectum.* (SG, spring-germinated plants; AG, autumn-germinated plants; CK, control treatment; N1, 35 kg ha^-1^yr^-1^; N2, 70 kg ha^-1^yr^-1^; N3, 140 kg ha^-1^yr^-1^. W30%N1, increased 30% precipitation on the basis of the current rainfall, and then increase 35 kg ha^-1^yr^-1^; W50%N1, increased 50% precipitation on the basis of the current rainfall, and then increase 35 kg ha^-1^yr^-1^).

In the years 2022 and 2023, this study established four nitrogen deposition levels: CK (control treatment, no nitrogen increased), N1 (simulating the nitrogen deposition rate of 35 kg ha^-1^yr^-1^ in Fukang City), N2 (simulating twice the future nitrogen deposition rate of 70 kg ha^-1^yr^-1^), and N3 (simulating an extreme nitrogen deposition rate of 140 kg ha^-1^yr^-1^). According to the nitrogen application method by Zhou et al. (2014) [33], nitrogen is applied twice a year, once in late October (before winter snowfall) and once in late March (during snowmelt), with half of the annual amount (50% nitrogen) applied each time. When applying nitrogen (N), NH_4_NO_3_ and NH_4_Cl are mixed in water at a ratio of NH_4+_-N: NO_3-_-N = 2: 1. There is less water in solution and equivalent to 0.27% of the annual precipitation (calculated based on an annual precipitation of 150 mm; the resulting ecological effects can be ignore), and evenly sprayed onto the surface of a 1 m^2^ plot from top to bottom and from left to right using an accurate sprayer to spatial uniformity of nitrogen application, while the control group is sprayed with an equal amount of water. Within the *H. erectum* population, in order to avoid interspecific competition, we removed other plants from the plots during the experiment. The density of *H. erectum* plants in the field is 55 plants/m^2^ [29]，the number of plants was enough for the experiments. Therefore, in this experiment, each treatment group was arranged with 12 quadrats of 1 m × 1 m (5 quadrats for phenology observation, 6 quadrats for plant traits and biomass sampling, and 1 quadrat as spare). To avoid interference from other areas and within the plots, the plots were spaced 2 m apart, and a 0.5 m deep impermeable layer (plastic sheet) was buried 1 m away from the sample plots (Fig 2).

### The interaction experiment of water increment and nitrogen increment

In the years 2022 and 2023, two treatments of 30% water increase + 35 kg ha^-1^yr^-1^ (W30%N1, abbreviation W3N1) and 50% water increase + 35 kg ha^-1^yr^-1^ (W50%N1, abbreviation W5N1) were established. To ensure precise control of water treatment, the water treatment method is the same as nitrogen increase experiment. Each treatment was configured with 12 quadrats of 1 m × 1 m (5 quadrats for phenology observation, 6 quadrats for plant traits and biomass sampling, and 1 quadrat as spare). To avoid the influence among treatments, each treatment was separated by 2 m, and a 0.5 m deep impermeable layer (plastic sheet) was buried 1 m away from the sample plots (Fig 2). Increase a corresponding proportion of water based on the amount of precipitation after each precipitation, and carry on water-nitrogen interaction treatment (the number of water-nitrogen interaction treatment is consistent with the number of natural precipitation during the experimental period). The natural precipitation is counted by four rainfall collectors placed in the field (each rainfall collector comprises a 1 m × 1 m plastic with an area of 1 m^2^. The plastic is at a 30° angle to the ground. The plastic is fixed with four iron rods, and a bucket is placed at the lower right corner to collect the rainfall for calculating the precipitation).

## Sampling and statistical

### Phenology

From October to November 2022 and from March to April 2023, phenology was observed within 5 quadrats for each treatment. Beginning from seed germination, observations were made once a day until the plants died，record the emergence date, leafing date, first flowering date, peak flowering date, last flowering date, fruiting date, withering date, and the duration of flowering and the life history cycle.

### Survivorship

Within all the quadrats of the 12 treatments during spring- and autumn-germinated plants, from the emergence date to the withering date, the number of surviving plants in the quadrats was recorded every 7 d.

### Plant traits

When the plants maturity, the root length, plant height, leaf area, number of leaves, number of branches and number of seeds of all the plants (totaling 1080 plants) within each of the 6 quadrats (the remaining quadrats after phenology observation, totaling 72 quadrats) under each of the 6 treatments (12 treatments) for both spring- and autumn-germinated plants were measured, respectively.

### Reproductive allocation

During the fruiting date, when the legumes are mature (yellow and have not dehiscence) to collect all the fruits. Due to the difference in maturity periods among different treatments, the collecting dates for other plant organs are determined by the collecting date of the fruit. When excavating the plants, in order to ensure the integrity of the samples, the excavation was carried out with *H. erectum* as the center, with a radius of 20 cm and a depth of 50 cm as the standard (the length of the roots of *H. erectum* is about 30–40 cm), and all the plants (a total of 1080 plants) in the 72 quadrats (the remaining quadrats after phenology observation) under 12 treatments of spring- and autumn-germinated plants were taken back to the laboratory. In the laboratory, each plant was divided into four parts: root, stem, leaf and fruit. They were dried in a drying oven at 80 °C for 48 h, respectively, and the dry weight was determined. The precision of the balance used was 0.001, and the Sartorius BS210S electronic balance (0.001 g) was used for weighing. The total biomass is the total of roots, stems, leaves and fruits. The biomass allocation of roots, stems, leaves and fruits is shown in percentage:


Root allocation = (roots mass)/ (total mass)× 100 % (1)

Stem allocation = (stems mass)/ (total mass)× 100 % (2)

Leaf allocation = (leaves mass)/ (total mass)× 100 % (3)

Reproductive allocation = (reproductive mass)/ (total mass)× 100 % (4)

### Seed germination characteristics

From May 25th to June 17th, 2023, all the mature seeds within the quadrats of spring- and autumn-germinated plants under different treatments in the two seasons were collected and brought back to the laboratory. Beginning on June 22th, 2023, the seeds (30 seeds, with 4 replicates) were uniformly placed in petri dishes with a diameter of 90 mm and laid with 2 layers of moist filter paper (3 mL of distilled water), and the seed germination experiment was carried out under the alternating temperature conditions of 15/25 °C (12/12 h light/dark). Seed germination munbers were tested once every 24 h during the germination process for a total of 30 d. Seed germination is defined by the protrusion of the radicle by 2 mm extend the seed coat. Germinated seeds are removed, and the lost water is replenished with distilled water concurrently. Final percentage of germination (FPG) was estimated as follows: FPG = GN/SN (GN is the total number of germinating seeds, and SN is the number of viable seeds). After the seed germination, TTC staining method was used to test the vitality of ungerminated seeds.

### Statistical analyses

The difference of year, increased nitrogen and the water-nitrogen interaction treatment on plant survival rate, plant traits, biomass and seed germination were analyzed by one-way ANOVA. Based on the results of the multi-factor analysis of variance via ANOVA, we used the Turkey method to conduct multiple comparisons of the growth and biomass variables with significant differences to determine the significant differences among different treatments. All the data analyses were carried out in SPSS 24.0, and the graphs were plotted with Origin 2018 (Origin Lab, Northamptom, MA).

## Results

### Phenology

On October 29th, 2022, the autumn-germinated plants with CK, N1, N2, and N3 as well as the water-nitrogen interaction treatment (W3N1 and W5N1) all germinated on the same day. The spring-germinated plants also germinated simultaneously on April 5th, 2023. Phenology (leafing date, first flowering date, peak flowering date, last flowering date, fruiting date and withering date) of autumn-germinated plants with CK, nitrogen increase (N1, N2, N3), and the water-nitrogen interaction treatment (W3N1, W5N1) was earlier than that of spring-germinated plants. The advance time of the leafing date is the longest, about 5 months, and the other indicators is about 1–2 weeks. The life history of autumn-germinated plants was longer than that of spring-germinated plants, the extension periods with CK, N1, N2, N3, W3N1 and W5N1was 141–144 d, 140–144 d, 140–144 d, 139–144 d, 143–146 d, and 143–145 d, respectively (Table 1).

**Table 1:** Effects of increased nitrogen, and water-nitrogen interaction treatment on phenology of spring- and autumn-germinated plants of *Hypecoum erectum.*

Phenology		Emergence date (month-day)	Leafing date (month-day)	First flowering date (month-day)	Flowering duration (d)	Peak flowering date (month-day)	Last flowering date (month-day)	Fruiting date (month-day)	Withering date (month-day)	Life cycle (d)
CK	SG	3-30 ~ 4-5	4-6 ~ 5-12	5-8	20	5-15	5-27	5-23 ~ 6-10	6-2 ~ 6-15	65 ~ 72
	AG	10-29 ~ 11-10	11-11 ~ 5-6	5-1	24	5-9	5-24	5-15 ~ 5-30	5-25 ~ 6-10	209 ~ 213
N1	SG	3-30 ~ 4-5	4-6 ~ 5-12	5-9	20	5-16	5-28	5-22 ~ 6-11	6-3 ~ 6-15	66 ~ 72
	AG	10-29 ~ 11-11	11-11 ~ 5-8	5-1	24	5-9	5-24	5-16 ~ 5-31	5-26 ~ 6-10	210 ~ 212
N2	SG	3-30 ~ 4-5	4-6 ~ 5-14	5-8	20	5-16	5-27	5-23 ~ 6-10	6-3 ~ 6-15	66 ~ 72
	AG	10-29 ~ 11-11	11-11 ~ 5-8	5-2	25	5-10	5-26	5-16 ~ 5-31	5-26 ~ 6-10	210 ~ 212
N3	SG	3-30 ~ 4-4	4-5 ~ 5-12	5-7	20	5-14	5-26	5-22 ~ 6-9	6-2 ~ 6-15	65 ~ 73
	AG	10-29 ~ 11-11	11-11 ~ 5-8	5-1	24	5-9	5-24	5-16 ~ 5-31	5-25 ~ 6-10	209 ~ 212
W30%N1	SG	3-30 ~ 4-7	4-8 ~ 5-13	5-9	21	5-18	5-29	5-25 ~ 6-13	6-3 ~ 6-15	66 ~ 70
	AG	10-29 ~ 11-10	11-11 ~ 5-7	5-4	25	5-12	5-28	5-17 ~ 6-1	5-28 ~ 6-10	212 ~ 213
W50%N1	SG	3-30 ~ 4-7	4-8 ~ 5-14	5-9	22	5-18	5-30	5-25 ~ 6-13	6-3 ~ 6-15	66 ~ 70
	AG	10-29 ~ 11-10	11-11 ~ 5-8	5-4	25	5-12	5-28	5-17 ~ 6-1	5-27 ~ 6-10	211 ~ 213

**Note:** SG, spring-germinated plants; AG, autumn-germinated plants; CK, control treatment; N1, 35 kg ha^-1^yr^-1^; N2,70 kg ha^-1^yr^-1^; N3, 140 kg ha^-1^yr^-1^. W30%N1, increased 30% precipitation on the basis of the current rainfall, and then increase 35 kg ha-1yr-1; W50%N1, increased 50% precipitation on the basis of the current rainfall, and then increase 35 kg ha^-1^yr^-1^.

### Survivorship

**Fig 3:**
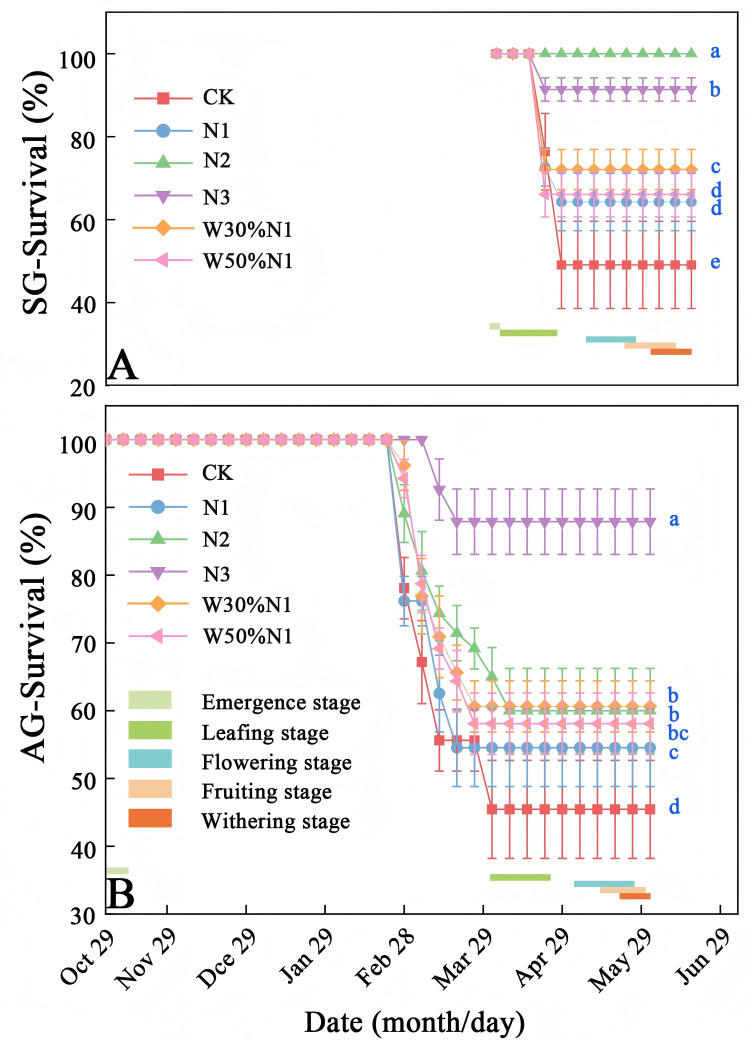
Survival of spring- and autumn-germinated plants of *Hypecoum erectum* in the treatment of increased nitrogen and water-nitrogen interaction treatment. (SG, spring-germinated plants; AG, autumn-germinated plants. CK, control treatment; N1, 35 kg ha^-1^yr^-1^; N2, 70 kg ha^-1^yr^-1^; N3, 140 kg ha^-1^yr^-1^. W30%N1, increased 30% precipitation on the basis of the current rainfall, and then increase 35 kg ha^-1^yr^-1^; W50%N1, increased 50% precipitation on the basis of the current rainfall, and then increase 35 kg ha^-1^yr^-1^). Different lowercase letters indicate significant differences among increased nitrogen and water-nitrogen interaction treatment (CK, N1, N2, N3, W30%N1 and W50%N1) for SG and AG (*p* < 0.05).

Survival rate of spring- and autumn-germinated plants (CK) significantly decreased on April 20th, 2023 and March 4th, 2023, respectively. Survival rate of autumn-germinated plants (45%) was lower than that of spring-germinated plants (49%) (Fig 3A–B). Nitrogen addition significantly improved the survival rate of both spring- and autumn-germinated plants (*p* < 0.05, ANOVA). Survival rate of spring-germinated plants with N1, N2, and N3 were 64%, 100%, and 91%, respectively; those of autumn-germinated plants with N1, N2, and N3 were 55%, 60%, and 88%, respectively (Fig 3A-B). Water-nitrogen interaction treatment significantly increased the survival rate of both spring- and autumn-germinated plants (*p* < 0.05, ANOVA). Survival rate of spring-germinated plants with W3N1 and W5N1 were 72% and 66%, respectively; and the survival rate of autumn-germinated plants W3N1 and W5N1 were 61% and 58%, respectively (Fig 3A-B).

### Plant traits

**Fig 4:**
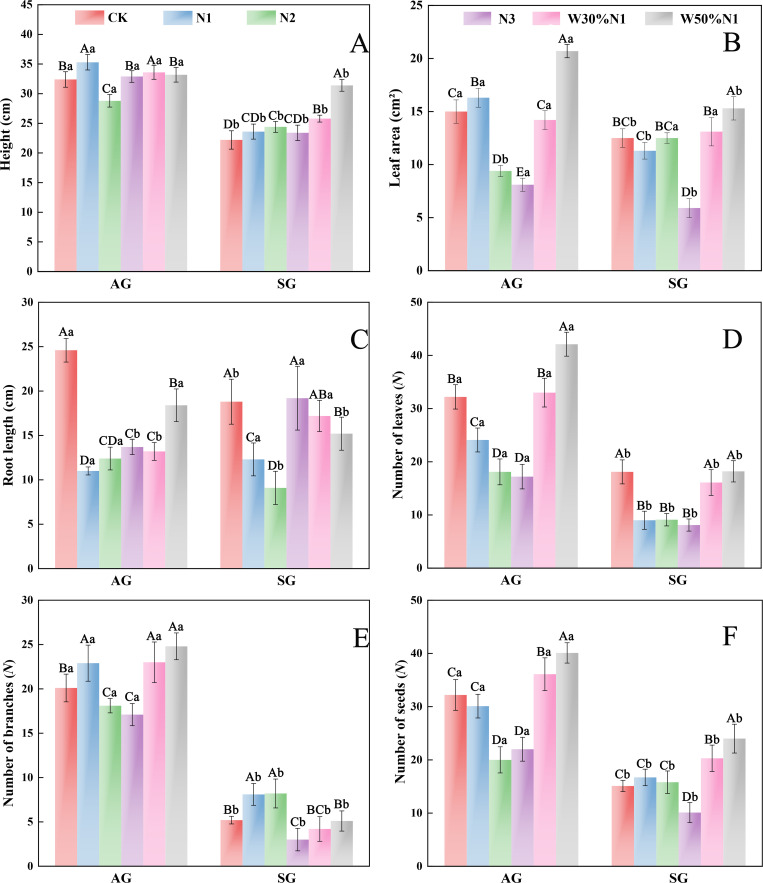
Effects of increased nitrogen and water-nitrogen interaction treatment on plant traits of spring- and autumn-germinated plants of *Hypecoum erectum.* (AG, autumn-germinated plants; SG, spring-germinated plants; CK, control treatment; N1, 35 kg ha^-1^yr^-1^; N2, 70 kg ha^-1^yr^-1^; N3, 140 kg ha^-1^yr^-1^. W30%N1, increased 30% precipitation on the basis of the current rainfall, and then increase 35 kg ha^-1^yr^-1^; W50%N1, increased 50% precipitation on the basis of the current rainfall, and then increase 35 kg ha^-1^yr^-1^). Different lowercase letters indicate significant differences between SG and AG at the same increased nitrogen or water-nitrogen interaction treatment (*p* < 0.05). Different uppercase letters indicate significant differences among increased nitrogen and water-nitrogen interaction treatment (CK, N1, N2, N3, W30%N1 and W50%N1) for SG and AG (*p* < 0.05).

With the increase in N (N1, N2), there was no significant differences in the leaf area and seed number of spring-germinated plants (*p* > 0.05, ANOVA), while the root length and the number of leaves significantly decreased (*p* < 0.01, ANOVA), and the number of branches significantly increased (*p* < 0.01, ANOVA). When the nitrogen increase was N3, there was no significant difference in plant height and root length (*p* > 0.05, ANOVA), while leaf area, leaf number, branch number and seed number all significantly decreased (*p* < 0.01, ANOVA). With the increase of nitrogen (N2, N3), the leaf area, root length, leaf number, branch number and seed number of autumn-germinated plants all significantly decreased (*p* < 0.01, ANOVA). When the nitrogen increase is N1, plant height, leaf area and the number of branches were all significantly increased (*p* < 0.01, ANOVA), while root length and the number of leaves were significantly decreased (*p* < 0.01, ANOVA) (Fig 4). The water-nitrogen interaction treatment (W3N1, W5N1) significantly increased the plant height and seed number of spring-germinated plants, but had no significant difference on the number of leaves and branches (*p* > 0.05, ANOVA). The water-nitrogen interaction treatment (W3N1, W5N1) significantly increased the number of branches and seeds of autumn-germinated plants and decreased the root length (*p* < 0.01, ANOVA) (Fig 4). Therefore, excessive nitrogen decreased the seed reproductive ability of spring- and autumn-germinated plants, after the water-nitrogen interaction treatment, the seed reproduction ability of the offspring was improved to ensure the successful reproduction of the population.

### Biomass accumulation and allocation

**Fig 5:**
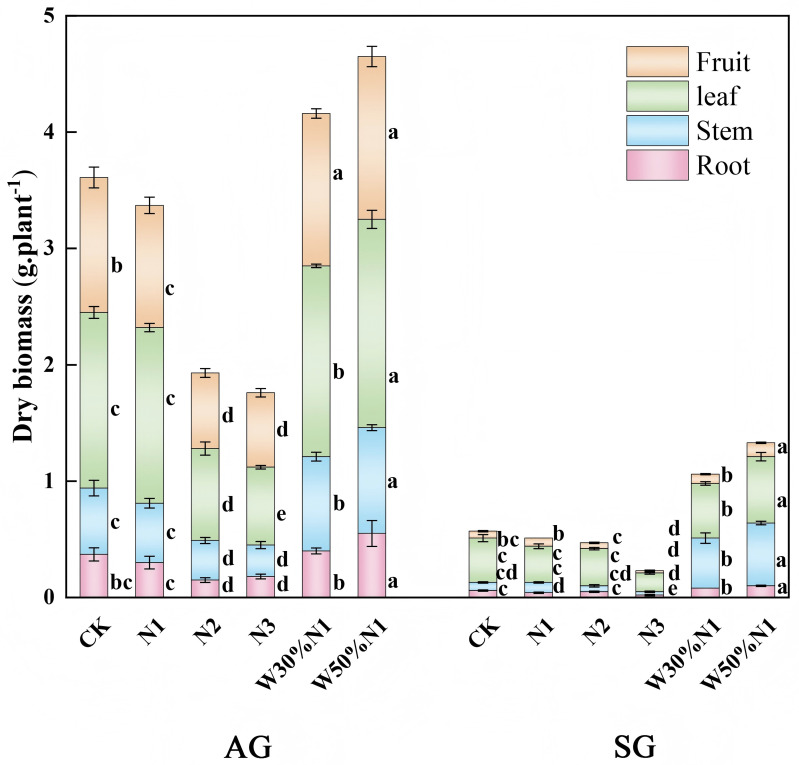
Effects of increased nitrogen and water-nitrogen interaction treatment on dry biomass of spring- and autumn-germinated plants of *Hypecoum erectum.* (Autumn plants, AG; spring plants, SG; control treatment, CK; 35 kg N ha-1 yr-1, N1; 70 kg N ha-1 yr-1, N2: 140 kg N ha-1 yr-1, N3; The precipitation increased by 30%, increase 35 N ha-1 yr-1, W30% N1; the precipitation increased by 50%, increase 35 N ha-1 yr-1,W50%N1). At present, there are different values observed in all processing, different lowercase letters indicate that there are significant differences (*p* < 0.05) in the effects of nitrogen increaseition and water-nitrogen interaction treatment (CK, N1, N2, N3, W30%N1 and W50%N1) on SG and AG, different uppercase letters indicate significant differences (*p* < 0.05) between SG and AG under the same nitrogen increaseition or water-nitrogen interaction treatment.

Among the six treatments (CK，N1, N2, N3, W3N1, W5N1), the total dry weight biomass of autumn-germinated plants was significantly higher than that of spring-germinated plants (*p* < 0.01, ANOVA). Increase of N (N1, N2, N3) decreased the biomass accumulation of spring- and autumn-germinated plants. Dry weight biomass of autumn-germinated plants decreased from CK (3.6 g) to N1 (3.37 g), N2 (1.93 g) and N3 (1.76 g). The dry weight biomass of spring-germinated plants decreased from CK (0.56 g) to N1 (0.51 g), N2 (0.48 g), and N3 (0.24 g). However, the water-nitrogen interaction treatment (W3N1, W5N1) increased the biomass accumulation of both spring- and autumn-germinated plants. The dry weight biomass of autumn-germinated plants from CK (3.6 g) to W3N1 (4.16 g) and W5N1 (4.65 g). The dry weight biomass of spring-germinated plants increased from CK (0.56 g) to W3N1 (1.05 g) and W5N1 (1.33 g) (Fig 5). During the growth and development of spring- and autumn-germinated plants, nitrogen interact with water to promote its growth and development.

**Fig 6:**
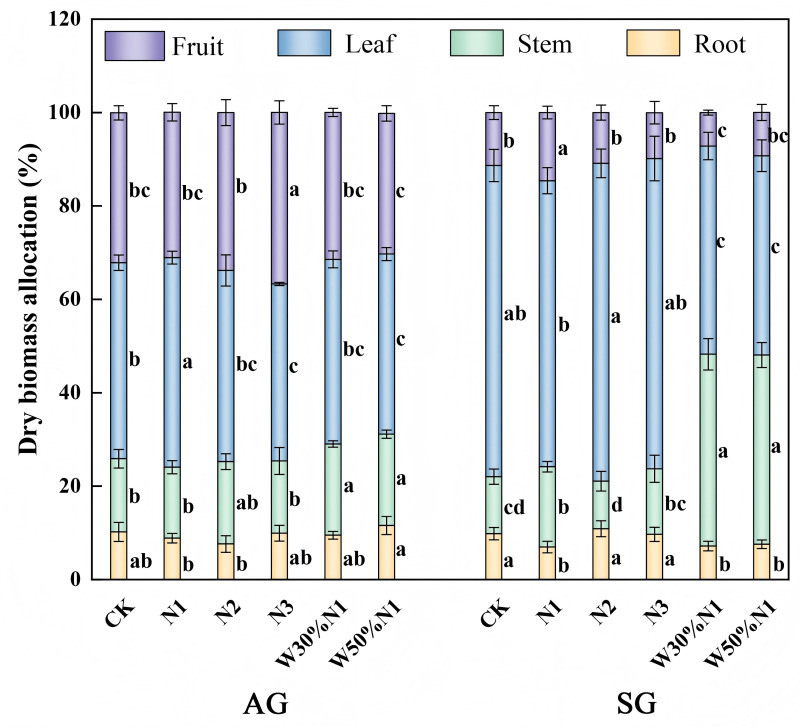
Effects of increased nitrogen and water-nitrogen interaction treatment on dry biomass allocation of spring- and autumn-germinated plants of *Hypecoum erectum.* (Autumn plants, AG; spring plants, SG; control treatment, CK; 35 kg N ha^-1^ yr^-1^, N1; 70 kg N ha^-1^ yr^-1^, N2: 140 kg N ha^-1^ yr^-1^, N3; The precipitation increased by 30%, increase 35 N ha^-1^ yr^-1^, W30% N1; the precipitation increased by 50%, increase 35 N ha^-1^ yr^-1^,W50%N1). At present, there are different values observed in all processing, different lowercase letters indicate that there are significant differences (*p* < 0.05) in the effects of nitrogen increaseition and water-nitrogen interaction treatment (CK, N1, N2, N3, W30%N1 and W50%N1) on SG and AG.

With the increase of nitrogen (N1), spring-germinated plants allocated more biomass to stems and fruits, while decreasing the biomass allocation to roots, and have no significant difference on the allocation to leaves (*p* > 0.05, ANOVA) (Fig 6). Autumn-germinated plants allocated more biomass to leaves, and show no significant difference on the allocation to roots, stems and fruits (*p* > 0.05, ANOVA). With the increased of nitrogen (N2), there was no significant difference on the biomass allocation of both spring- and autumn-germinated plants (*p* > 0.05, ANOVA). With the increase of nitrogen (N3), there was no significant difference on the biomass allocation of spring-germinated plants (*p* > 0.05, ANOVA); More biomass would be allocated to fruits, and the biomass allocation to leaves would decreased, while there was no significant difference on the allocation to roots and stems for autumn-germinated plants (*p* > 0.05, ANOVA). Water-nitrogen interaction treatment (W3N1, W5N1) result in an increase in the biomass allocation of spring-germinated plants to stems, while a decrease in the biomass allocation to roots, leaves and fruits. The biomass allocation to stems increased, the biomass allocation to leaves decreased, and there was no significant difference on the allocation to roots and fruits for autumn-germinated plants (*p* > 0.05, ANOVA). Spring- and autumn-germinated plants allocated more biomass to reproductive growth with nitrogen increased. The water-nitrogen interaction treatment had no effect on reproduction and growth.

### Seed germination characteristics

**Fig 7:**
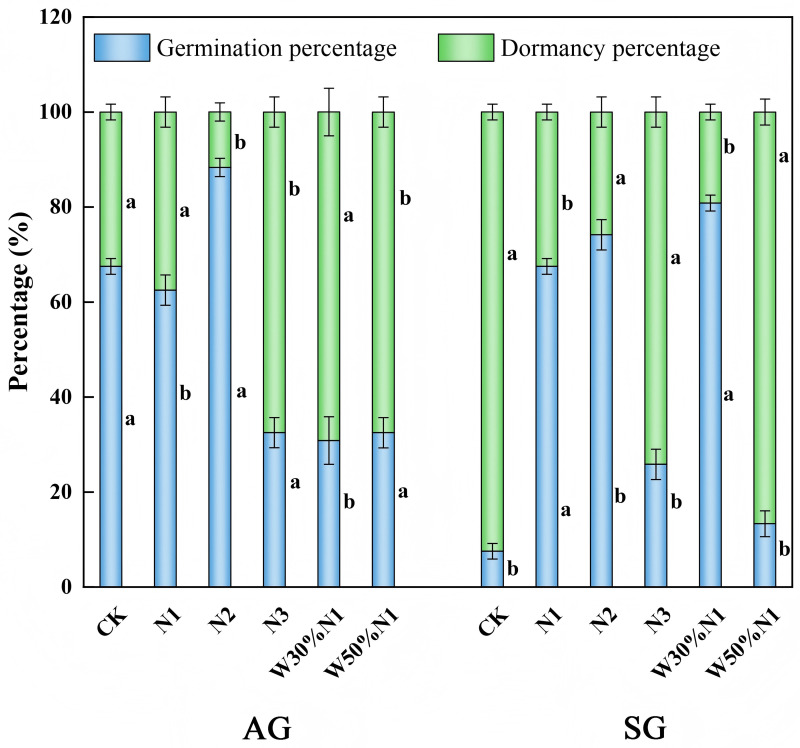
Effects of increased nitrogen and water-nitrogen interaction treatment on offspring germination from spring- and autumn-germinated plants of *Hypecoum erectum* incubated in light/dark at 25/15 °C. (Autumn plants, AG; spring plants, SG; control treatment, CK; 35 kg N ha^-1^ yr^-1^, N1; 70 kg N ha^-1^ yr^-1^, N2: 140 kg N ha^-1^ yr^-1^, N3; The precipitation increased by 30%, increased 35 N ha^-1^ yr^-1^, W30% N1; the precipitation increased by 50%, increase 35 N ha^-1^ yr^-1^,W50%N1). At present, there are different values observed in all processing, different lowercase letters indicate that there are significant differences (*p* < 0.05) in the effects of increased nitrogen and water-nitrogen interaction treatment (CK, N1, N2, N3, W30%N1 and W50%N1) on SG and AG).

In the control (CK), the germination rate of the seed from spring- and autumn-germinated plants were 67.51% and 7.53%, respectively (Fig 7). With the nitrogen (N1, N2, N3) increased, the germination rate of spring- and autumn-germinated plants offspring both showed an increase at first and then decreased, with the highest nitrogen inhibiting seed germination. Meanwhile, after the nitrogen increases, spring- and autumn-germinated plants will produce more non-dormant seeds to ensure that they can quickly occupy the population after the seeds mature in the same year. After the water-nitrogen interaction treatment (W3N1, W5N1), the germination rate of the spring-germinated seedlings decreased, from 67.51% (CK) to 30.84% (W3N1) and 32.50% (W5N1), while the germination rate of the autumn-germinated seedlings increased from 7.53% (CK) to 80.83% (W3N1). The water-nitrogen interaction treatment is more suitable for the germination and reproduction of the autumn-germinated seedlings. At the same time, the water-nitrogen interaction treatment resulted in a large number of dormant seeds that germinated the following year, thus spreading the risk of extinction that might be caused by once concentrated germination in adverse environment.

## Discussion

As climate change and global change continue to intensify, precipitation and nitrogen deposition in temperate desert ecosystems are undergoing dramatic changes, while water and nitrogen are the main limiting factors in desert ecosystems. The changes in precipitation and the continuous increase in nitrogen deposition have had a significant influence on plant growth and ecosystem nutrient cycling [34,35]. Water and nitrogen have a significant impact on the stability of desert ecosystems, significantly promoting plant growth, increasing biodiversity and biomass, promoting microbial activity, maintaining soil structure stability, and alleviating drought stress [36,37]. At the same time, it can also to disrupt ecological balance, cause soil nutrient imbalance, pollute soil and water resources, and advance desertification processes [38]. In the next 30 years, precipitation in the northwest region of China is expected to increase by about 30%, and the increase could reach 50% in extreme precipitation years [22]. The nitrogen deposition rate in the city of Fukang, the nearest city to the southern edge of the Gurbantunggut Desert, is 35.4 kg ha^-1^yr^-1^, and it will continue to increase by more than twice its current level in the future [32]. This study is the first to use the *H. erectum*, which grown in spring and autumn in the Gurbantunggut Desert, as an experimental material to investigate the effects of water-nitrogen interaction treatment on its life history and analyze the response of the plant to global change in both season.

Autumn-germinated plants effectively cope with winter damage through physiological, molecular mechanism, morphology, and ecological adaptation mechanisms to ensuring smooth recovery of growth in spring [39,40,41]. In the control, the survival rate of autumn-germinated plants was lower than that of spring-germinated plants, mainly due to that autumn-germinated plants suffered damage to their roots, stems, and leaves during the five-month long winter at low temperatures, while spring-germinated plants were able to avoid dying in winter. Nitrogen deposition and water are the important factors that affect the rhythm and phenology of plant growth [42]. Because nitrogen is a primary component of plant organism and plays a crucial role in the establishment of plant seedlings, as well as the growth of stems and leaves, and water as a solvent for nitrogen to promote growth ability by increasing nitrogen absorption efficiency and improving cellular water balance, so the survival rate of spring- and autumn-germinated plants was significantly increased after the increased of nitrogen and water-nitrogen interaction treatment. The absorption of nitrogen and water by plant roots significantly influences the growth and development of plants. Some studies have shown that as the water increases, the survival rate was increased, but as the water content continues to increase, it actually decreases the survival rate [43]. The survival rate of W3N1 is higher than that of W5N1 of spring- and autumn-germinated plants. This may be due to increased precipitation leading to soil hypoxia, nitrogen loss, mechanical damage, and poor plant adaptability. Meanwhile, there is a species specific response to the impact of precipitation changes on plant survival rates, as precipitation and other environmental factors work together to affect plant survival rates.

Allocation of plant resources is a form of adaptive selection, in which plants accumulate and distribute resources for the allocation to survival, growth, reproduction, and defense [44]. Annual herbaceous plant exhibits a positive correlation between plant traits and biomass accumulation, with larger individual plants accumulating higher biomass [45,24]. The availability of resources for plants varies throughout the growing season, and plants increase their fitness by adjusting the allocation of resources between growth and reproduction to adapt to these changes [46,47]. In this study, with the increased of nitrogen (N3), the number of leaves, leaf area, branch number and seed number of spring- and autumn-germinated plants all decreased. There was a no significant diffierence on plant height. Therefore, the biomass accumulation of spring- and autumn-germinated plants decreased with the increased of nitrogen. Chen et al. (2019) [2] found that with increasing nitrogen, most of the biomass accumulation of *Erodium oxyrhinchum* reduced, which is consistent with our research results. We hypothesize that the decreased in biomass accumulation after nitrogen increased may be due to the significant difference in precipitation over 5 mm in the study area between 2019 (56.23 mm) and 2018 (80.6 mm). Therefore, low soil moisture can lead to poor nitrogen uptake by roots. Research had found that an increase in nitrogen decreased in biomass accumulation and species diversity of herbaceous plants in arid and semi-arid desert, and change the species composition, and lead to ecosystem degradation [48,49]. In the Gurbantunggut Desert, artificial snow led to an increase in nitrogen which promoted the growth of ephemeral plants. Conversely, after artificial snow reducted, an increase in nitrogen inhibited the growth of ephemeral plants [40]. Nitrogen and water are two factors for plant growth, with water also being an essential prerequisite for efficient nitrogen utilization by plants. In this study, water-nitrogen interaction treatment significantly increased in biomass accumulation of both spring- and autumn-germinated seedlings, providing further support for the role of water in promoting nitrogen absorption. Due to the consistent resource selection and utilization mechanisms of the same plant, most plant traits and biomass of spring- and autumn-germinated plants show similar response when nitrogen increased and water-nitrogen interaction treatment. However, there was some difference due to the difficult life cycles. Therefore, spring-germinated plants show more sensitive to climate change than autumn-germinated plants.

Water can dissolve and migrate nitrogen, and the synergistic effect of water and nitrogen can avoid excessive nitrogen, optimize and promote balanced plant growth, ensure photosynthesis in aboveground parts, promote the opening and closing of plant stomata and root development, improve water use efficiency, and alleviate soil drought pressure [50,51]. Nitrogen and water increased changes the resource accumulation in the soil, and influenced plant growth and biomass allocation [52]. The response mechanisms of plants to increased nitrogen and water may difference by species growth specificity [53,54]. In this study, as nitrogen increased, spring- and autumn-germinated plants allocated more biomass to fruits, which provides nutritional for producing large seeds and will be beneficial to the successful reproduction of the *H. erectum* population in the desert extreme environment. Plants adjust biomass allocation to different organs under appropriate conditions to maintain relative stability [55]. In the study of water-nitrogen interaction treatment, biomass allocation among four organs of spring- and autumn-germinated plants were consistent, which is consistent with the results of four ephemeral plants of Trigonella [56]. Therefore, biomass allocation is an important strategy for population continuity in a constantly changing environment.

Reproductive strategy involves optimizing allocation strategies to adapt to changing environment, and enhancing the fitness of plants [46]. The reproduction allocation of plants are the result of life history evolution and also reflect their adaptability to the current environment [57]. Fruit allocation under extreme environmental conditions is an important strategy for plants to adapt to the environment, optimize resource utilization, and ensure reproduction and population continuity [58]. In this study, the water-nitrogen interaction treatment significantly delayed the reproductive growth and life cycle of spring- and autumn-germinated plants. After a delayed reproductive growth and life cycle, the nutritional growth of the plant is also prolonged, ensuring that the plant produces more seeds during the reproductive stage and improving population survival through seed production in extreme environments [59]. This conclusion is consistent with previous research on *Plantago minuta*, indicating that both nutritional growth and reproductive growth play important roles in seed development [24]. Therefore, nitrogen deposition and water-nitrogen interaction treatment will both improve the establishment of seedlings and plant growth of ephemeral plants in arid and semi-arid ecosystems in the future [60].

Seed fitness is the capacity of seeds to germinate and produce seedlings in suitable environments. Dormancy is one of the important mechanisms for seeds to successfully enter the seedling stage under favorable germination [61]. Seed as the main way for plants to continue their offspring, which have an impact on the survival rate and genetic variation of offspring through seed dormancy, which affects plant communities [62]. Moreover, seed dormancy time, seed quantity, and quality changes can inevitably affect the growth and survival of offspring [63]. Environmental changes affect seed dormancy and germination, which in turn affect seedling development. Seed dormancy is an adaptation to the environment that helps seeds to spend adverse conditions such as drought and cold, and avoid overcrowding in the next year, it is a result of long-term natural selection [64]. In water and nitrogen experiments on *Sinapis arvensis*, it was found that the dormancy rate of their offspring seeds gradually increased with the increase of water and nitrogen [65]. In this study, a large number of dormant seeds were produced by both spring- and autumn-germinated plants with the W5N1 treatment. This germination mechanism decreased the risks with the harsh environment of the Gurbantunggut Desert by spreading out the seed germination time, thus avoiding potential population extinction by once concentrated germination. Due to extreme drought, high temperatures, and resource scarcity in desert environments, non-dormant seeds can quickly germinate and grow, completing their life cycle under short favorable conditions, and thus survive and reproduce in harsh environments [66]. In the results of this study, it was observed that the non-dormant seeds of spring- and autumn-germinated plants showed a significant increased with the nitrogen (N2) increased. Seed germinated at an appropriate time and space is an important characteristics of life history.

## Conclusion

This study systematically investigated the response mechanism of the life history of *H. erectum*, an ephemeral plant in early spring in the Gurbantunggut Desert, to increase nitrogen and water-nitrogen interaction treatment. Nitrogen increase and water-nitrogen interaction treatment significantly improved the survival rate, prolonged the reproductive and life cycle, and increased seed numbers of spring- and autumn-germinated plants, which improve the reproductive ability and effectively avoid death which caused by environment and population competition. Simultaneously, the biomass accumulation of spring- and autumn-germinated plants decreased with increasing nitrogen, while it increased with the increase of water-nitrogen interaction treatment. Spring- and autumn-germinated plants allocate more biomass to reproductive organs to ensure the successful reproduction of *H. erectum* in extreme desert environments. Spring- and autumn-germinated plants produce more non-dormant seeds by increasing nitrogen, which can quickly occupy the population in next year. Water-nitrogen interaction treatment produces more dormant seeds to spread the risk of population extinction caused by once concentrated germination.

## Supporting information

S1 Graph Graph 1 original data. (XLS)

S1 Fig Figure 3 original data. (XLS)

S2 Fig Figure 4 original data. (XLS)

S3 Fig Figure 5 original data. (XLS)

S4 Fig Figure 6 original data. (XLS)

S5 Fig Figure 7 original data. (XLS)
